# The suitability of common metrics for assessing parotid and larynx autosegmentation accuracy

**DOI:** 10.1120/jacmp.v17i2.5889

**Published:** 2016-03-08

**Authors:** William J. Beasley, Alan McWilliam, Adam Aitkenhead, Ranald I. Mackay, Carl G. Rowbottom

**Affiliations:** ^1^ Institute of Cancer Sciences The University of Manchester Manchester UK; ^2^ Christie Medical Physics and Engineering The Christie NHS Foundation Trust Manchester UK; ^3^ Physics Department The Clatterbridge Cancer Centre NHS Foundation Trust Bebington UK

**Keywords:** automatic segmentation, adaptive radiotherapy, treatment planning, head and neck

## Abstract

Contouring structures in the head and neck is time‐consuming, and automatic segmentation is an important part of an adaptive radiotherapy workflow. Geometric accuracy of automatic segmentation algorithms has been widely reported, but there is no consensus as to which metrics provide clinically meaningful results. This study investigated whether geometric accuracy (as quantified by several commonly used metrics) was associated with dosimetric differences for the parotid and larynx, comparing automatically generated contours against manually drawn ground truth contours. This enabled the suitability of different commonly used metrics to be assessed for measuring automatic segmentation accuracy of the parotid and larynx. Parotid and larynx structures for 10 head and neck patients were outlined by five clinicians to create ground truth structures. An automatic segmentation algorithm was used to create automatically generated normal structures, which were then used to create volumetric‐modulated arc therapy plans. The mean doses to the automatically generated structures were compared with those of the corresponding ground truth structures, and the relative difference in mean dose was calculated for each structure. It was found that this difference did not correlate with the geometric accuracy provided by several metrics, notably the Dice similarity coefficient, which is a commonly used measure of spatial overlap. Surface‐based metrics provided stronger correlation and are, therefore, more suitable for assessing automatic segmentation of the parotid and larynx.

PACS number(s): 87.57.nm, 87.55.D, 87.55.Qr

## I. INTRODUCTION

Intensity‐modulated radiotherapy (IMRT) and volumetric‐modulated arc therapy (VMAT) are capable of creating highly conformal treatment plans, with steep dose gradients providing efficient organ at risk (OAR) sparing.^1^, ^2^ IMRT has been shown to benefit patients in the head and neck,^3^, ^4^, ^5^ with the PARSPORT trial (Institute of Cancer Research, London, UK) demonstrating reduced incidence of xerostomia in patients treated with parotid‐sparing IMRT relative to those treated with conformal radiotherapy.^(6)^ However, in order to realize the benefits afforded by IMRT, accurate delineation of targets and normal structures is essential.^(7)^

Contouring in the head and neck is time‐consuming and labor‐intensive,^8^, ^9^ but automatic segmentation has shown potential to reduce interobserver variation and improve efficiency by reducing the time required for outlining.^7^, ^10^, ^11^ This is of particular benefit to adaptive radiotherapy (ART), and there has therefore been much interest in automatic segmentation, with several algorithms having been assessed for accuracy.^7^, ^11^, ^12^, ^13^

**Figure 1:**
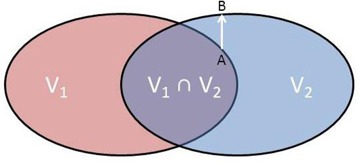
DSC and DTA. DSC measures the spatial overlap between two volumes, and DTA describes the shortest distance between two surfaces for a specific point.

In such studies, the accuracy of automatic segmentation algorithms has been assessed by measuring geometric agreement between automatically generated structures and ‘ground truth’ structures provided by manual delineation. A wide variety of metrics have been reported in the literature, and can be broadly separated into volume‐based and surface‐based metrics. Volume‐based metrics, such as the Dice similarity coefficient (DSC), which measures the spatial overlap of two volumes (see Fig. 1), and the conformity index (CI), which measures the relative difference in volumes, are commonly used.^15^, ^16^, ^17^ Whilst these metrics are relatively simple to understand, they are difficult to interpret and are sensitive to the volumes of the structures being assessed.^18^, ^19^ Surface‐based metrics provide a quantitative measure of the concordance of two surfaces and are typically based on distance‐to‐agreement (DTA). DTA is calculated by computing the minimum distance from a point on a reference surface to any point on a target surface (see Fig. 1), which is repeated for all points on the reference surface. From this, a DTA histogram can be produced. Several different metrics can be derived from this DTA histogram and some of the most commonly reported include the mean‐ and maximum‐DTA^14^, ^20^ and the 95%‐Hausdorff distance (95%‐HD),^21^, ^22^ which is defined as the 95th percentile of the DTA histogram. Although these metrics provide a measure of the distance between two structures, they too can be difficult to translate into clinical relevance.^(19)^

To parallel the term ‘geometric accuracy’, which quantifies the spatial agreement of two different structures, we introduce the term ‘dosimetric accuracy’ to quantify the difference in dose between two structures within a given dose distribution. In the case of automatic segmentation, the goal is to create automatically generated structures with high geometric accuracy relative to the ground truth. Similarly, within the context of treatment planning and evaluation, in which an automatically generated contour might be used for treatment planning, it is also important that the dose reported to an automatically generated contour agrees with the dose reported to the corresponding ground truth structure.^(23)^ Erroneous dose reporting may ultimately lead to a suboptimal plan.

With such a variety of spatial metrics available, there is no consensus as to the most suitable metric for assessing geometric accuracy.^18^, ^24^ As both the geometric and dosimetric accuracy are important for treatment planning and evaluation, it can be argued that suitable spatial metrics are those that provide results related to dosimetric accuracy.^(25)^ A geometrically accurate contour, as measured with a suitable spatial metric, should therefore be reflected in a small dosimetric difference, and vice versa.

In the present study, the geometric and dosimetric accuracy are measured for the parotid and larynx in head and neck VMAT treatment planning. The relationship between the geometric and dosimetric accuracy is measured, thus identifying suitable spatial metrics.

## II. MATERIALS AND METHODS

Five clinicians outlined the parotids and larynx for 10 head and neck cancer patients. These contours were created as part of a recent study at our institution assessing the geometric accuracy of a commercial automatic segmentation algorithm. The contouring has previously been described,^(14)^ but is briefly outlined here. Contouring was performed according to locally agreed protocols, and all observers contoured the structures independently, with access to the same clinical information. The observers were free to adjust the windowing and level according to personal preference. For each structure, the five clinician contours were combined into a single ground truth contour using the simultaneous truth and performance level estimation (STAPLE) algorithm, which computes a probabilistic estimate of the ground truth from multiple segmentations of the same structure.^(26)^ The resulting STAPLE contours were used as the reference standard (i.e., the ground truth) against which automatic contours were compared.

### A. Dosimetric and geometric accuracy

For each patient, dual‐arc 6 MV Elekta VMAT plans (Elekta, Stockholm, Sweden) were retrospectively created using the Philips Pinnacle^3^ v9.6 treatment planning system (Philips Radiation Oncology Systems, Andover, MA), according to standard departmental protocols (see Table 1). Planning target volumes (PTVs) were created from a uniform 4 mm expansion of relevant clinical target volumes (CTVs), which had been drawn at the point of initial treatment; automatic segmentation of target volumes was not investigated. Automatically generated normal structures were created using the Philips Smart Probabilistic Contouring Engine (SPICE) software. These automatically generated contours were then used directly in the plan optimization, with a 5 mm uniform margin applied to the spinal cord and brainstem to create planning organ at risk volumes (PRVs). The STAPLE contours for the parotids and larynx were imported into the treatment plan and the mean doses to these structures (the ‘true’ doses) were compared to those of the corresponding automatically generated contours. The mean dose was used as this is the dosimetric parameter of interest when assessing a treatment plan for these structures. The dosimetric accuracy was then defined as the percentage difference between the mean dose to the automatically generated and ground truth structures, relative to the dose to the ground truth structure.

In addition to measuring the dosimetric accuracy for the automatically generated structures, the difference in mean dose to the individual clinician contours relative to the true dose (dose to the STAPLE contour) was also measured for each patient. This provided a measurement of the dosimetric interobserver variation in mean dose for each patient, ultimately defining the range within which the dose to the automatically generated structure is acceptable.

**Table 1:** OAR dose constraints used for creating the VMAT plans

*OAR*	*Dose Constraint/cGy*
Spinal cord PRV	Max<4800
	$\text{Max}\;1\;{\text{cm}}^{3}<4500$
Brainstem PRV	Max<5400
	$\text{Max}\;1\;{\text{cm}}^{3}<5000$
Contralateral parotid	Mean<2600
Larynx	Mean<4500
Oral cavity	Mean<4500

A number of commonly used metrics were used to measure the geometric accuracy of the automatically generated contours relative to the ground truth structures, using an in‐house MATLAB script (MathWorks, Natick, MA). Two volume‐based metrics were investigated: the conformity index (CI), which is the ratio of the volumes of the two structures; and DSC, which is a measure of the spatial overlap of two structures, defined as $\text{DSC}=2(V_{1}\cap V_{2})/(|V_{1}|+|V_{2}|)$ (see Fig. 1). The centroid separation, which is the magnitude of the distance between the centers of mass of two structures, was also measured.

The other metrics were based on the surface agreement of two structures, and an in‐house MATLAB script was used to calculate a DTA histogram for each structure pair. DTA is defined for a particular point on a reference surface, A, as the shortest distance to any point on surface B (see Fig. 1). This is performed for each point on surface A, and a cumulative DTA histogram is created. From this DTA histogram, the mean and maximum DTA were measured, along with the 95%‐Hausdorff distance (95%‐HD), measuring the 95th percentile of the cumulative DTA histogram.

### B. Relationship between geometric and dosimetric accuracy

The correlation between the dosimetric accuracy and the different metrics was measured using the Pearson product‐moment correlation coefficient; the strength of the correlation indicated the strength of the relationship between the geometric and dosimetric accuracy.

## III. RESULTS

### A. Dosimetric and geometric accuracy

**Figure 2:**
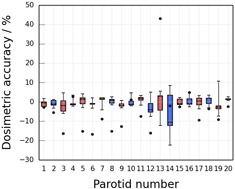
Dosimetric interobserver variation for the parotids. Box plot showing the interobserver variation in dosimetric accuracy relative to the STAPLE contours for the parotid glands. Red boxes indicate right hand parotid glands and blue boxes indicate left hand glands. The boxes indicate the interquartile range, the whiskers indicate the minimum and maximum variation, and the horizontal lines indicate the median accuracy of the five clinician contours. The mean dosimetric accuracy of the automatically generated contours is indicated by the circles.

**Figure 3:**
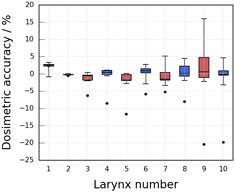
Dosimetric interobserver variation for the larynx. Box plot showing the interobserver variation in dosimetric accuracy relative to the STAPLE contours for the larynx. The boxes indicate the interquartile range, the whiskers indicate the minimum and maximum variation, and the horizontal lines indicate the median accuracy of the five clinician contours. The mean dosimetric accuracy of the automatically generated contours is indicated by the circles.

The mean dosimetric accuracy was measured to be −4.8±3.4% and −8.4±2.3% (dose to the automatically generated contours lower than that to the STAPLE contours) for the parotids and larynx, respectively. The uncertainties were estimated from the mean standard deviations in the interobserver variation in mean dose, which provides an estimate of the uncertainty in the dose delivered to the ground truth contours. Figures 2 and 3 show box plots of the dosimetric interobserver variation for the individual parotid and larynx structures, respectively. The boxes indicate the interquartile range and the whiskers indicate the maximum and minimum range of variation in mean dose to the five clinician‐drawn structures relative to the STAPLE contour (equal to the interobserver variation). The dosimetric accuracy of the automatically generated contours is also indicated for each structure by the black circles; it can be seen that the dose to the automatically generated contour was outside the dosimetric interobserver variation for 16 out of the 20 parotid glands and nine of the ten larynx contours. Note that parotid 13 (Fig. 2) has a dosimetric accuracy of +43%. This was caused by the gland's being in a region of low dose (mean doses of 376 cGy and 514 cGy to the STAPLE and automatically generated contour, respectively), resulting in a large relative difference in mean dose between the SPICE and STAPLE contours. Similarly, parotid 14 also received a low mean dose (approximately 500 cGy), so the interobserver variation was relatively large for this gland.

### B. Relationship between geometric and dosimetric accuracy

**Figure 4:**
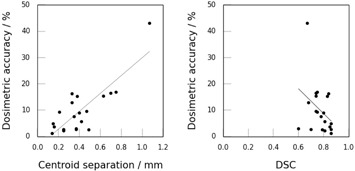
Scatter plots showing the relationship between dosimetric and geometric accuracy for the parotid. The left hand plot shows the relationship for the centroid separation (R=0.82), and the right hand plot shows the relationship for DSC (R=−0.35). Lines of best fit are also shown.

Table 2 shows the correlation coefficients between the dosimetric accuracy and the various metrics. There was no correlation between the volume‐based metrics (DSC and CI) and dosimetric accuracy for the parotids. Metrics based on surface agreement (DTA) showed statistically significant (p<0.05) correlations with dosimetric accuracy, with meanDTA and 95%‐HD showing strong correlation. The strongest correlate for the parotid was found to be the centroid separation. This can be seen in Fig. 4, which shows scatter plots of the dosimetric accuracy as a function of centroid separation (left hand plot), for which correlation was strong and statistically significant, and DSC (right hand plot), for which correlation was weak and not statistically significant (p>0.05).

Centroid separation did not correlate with dosimetric accuracy for the larynx, and weak correlation was observed for the volume‐based metrics. Strong correlation was observed for the surface‐based metrics.

**Table 2:** Correlation coefficients between the different metrics and the dosimetric accuracy

*Metric*	*Parotid*	*Larynx*
DSC	−0.35	−0.59 ^a^
CI	−0.33	0.58^a^
Centroid separation	0.82^a^	0.50
maxDTA	0.55^a^	0.60^a^
meanDTA	0.69^a^	0.64^a^
95%‐HD	0.61^a^	0.63^a^

a ^a^ Statistical significance at p<0.05.

## IV. DISCUSSION

Automatic segmentation will be an essential component of treatment planning and ART, and it is important to assess the geometric accuracy of automatic segmentation algorithms before clinical implementation. There are many widely accepted spatial metrics for assessing geometric accuracy, but there is no consensus as to the most appropriate metrics to use. For treatment planning and evaluation, it is also important that the dose to an automatically generated structure agrees with the dose to its corresponding ground truth structure. This work proposes that an appropriate spatial metric is one that correlates with dosimetric accuracy, and aims to identify spatial metrics suitable for assessing automatic segmentation accuracy of the parotid and larynx in the head and neck.

The results have indicated that several commonly used geometric metrics do not correspond to dosimetric accuracy and are not suitable for assessing automatic segmentation performance for certain OARs in the head and neck. Notably, it has been shown that DSC is a poor surrogate for dosimetric accuracy for the parotids. This is highlighted by the fact that the mean parotid DSC in this study was 0.77, which is generally considered to be clinically acceptable,^14^, ^27^ but the mean dose to the automatically generated structures was outside the range of dosimetric interobserver variation for 16 out of the 20 parotid glands investigated.

Although DSC and CI did not correlate with dosimetric accuracy for the parotids, the remaining metrics provided statistically significant correlations. The centroid separation, which is the magnitude of the distance between the centers of mass of the automatically generated and STAPLE contours, provided the strongest correlation. This is likely explained by the fact that the parotids are often in close proximity to the target volume and in the region of a unidirectional steep dose gradient, such that global differences in organ position have a large effect on dosimetric accuracy. In contrast, the centroid separation did not correlate with dosimetric accuracy for the larynx. This was likely caused by the fact that the larynx is often in the region of several dose gradients, and so a global shift of position does not necessarily change the mean dose.

The surface‐based metrics correlated with dosimetric accuracy for both the parotids and larynx, although the correlation for maxDTA was weaker than for meanDTA and 95%‐HD, probably due to the fact that a discrepancy in a single point on a surface does not necessarily have a large effect on the mean dose to that structure. Nevertheless, the correlation of the surface‐based metrics with dosimetric accuracy suggests that these metrics are suitable for assessing automatic segmentation accuracy.

Whilst there have been many studies reporting the geometric accuracy of various automatic segmentation algorithms,^7^, ^13^, ^14^, ^28^ there have been relatively few that have investigated the dosimetric effect of automatic segmentation uncertainties in head and neck IMRT.^25^, ^29^ Tsuji et al.^(25)^ investigated the dosimetric accuracy in 16 patients treated with head and neck IMRT. They compared the doses delivered to automatic and manual contours, and reported that the dosimetric differences were significant for the targets, but minor for the OARs. In contrast, Eiland et al.^(29)^ reported significant dosimetric differences between automatic and manual contours in seven head and neck IMRT plans, and concluded that automatic segmentation cannot yet replace manual delineation for treatment planning. This is in agreement with our findings, which show that the dose delivered to automatic contours is generally outside the range of interobserver variation.

The above studies measured the difference between dosimetric parameters for different structures in the head and neck for automatic and manual contours, using a single observer to define the ground truth. However, in our study, five clinicians outlined each structure, providing the interobserver variation in dose for individual patients. This enabled a more realistic assessment of the acceptability of automatic contours, as the dose to an automatically generated structure could be compared to the interobserver variation for the specific patient in question. Additionally, the use of a STAPLE volume provided a better estimate of the ground truth than for a single observer.^(26)^

Metrics suitable for assessing the geometric accuracy of automatic segmentation algorithms should be related to dosimetric accuracy, and there is no consensus as to which metrics are suitable for use in head and neck VMAT. Tsuji et al.^(25)^ investigated the relationship between geometric and dosimetric accuracy for two metrics: DSC and the overlap index (OI), which measures the proportion of the manual contour within the automatic contour. Although they reported correlation between GTV dosimetric agreement and the OI, there was no correlation for the OARs. This supports the results obtained in the present study, where the volume‐based metrics did not correlate with differences in mean dose; however, the authors did not investigate surface‐based metrics. Nelms et al.^(23)^ did investigate a surface‐based metric, the ‘linear penalty’, which is a modified DTA giving more weight to larger contour discrepancies. Although they reported that this metric was related to dosimetric accuracy, the linear penalty is not commonly used; nevertheless, this supports our findings that surface‐based metrics are suitable for measuring automatic segmentation accuracy.

In the present study, a single commercial automatic segmentation algorithm was used to generate automatic contours. It should be emphasized that the relationship between the geometric and dosimetric accuracy would be independent of the specific automatic segmentation algorithm used.

The present study used a small dataset of 10 head and neck patients to assess the relationship between dosimetric and geometric accuracy for the parotid and larynx. These structures are considered parallel organs, and the results cannot be extrapolated to serial organs in the head and neck, such as the spinal cord and brainstem. The location of these structures relative to typical dose gradients, as well as the fact that the dosimetric parameter of interest is the maximum dose, means that further work is required to determine which metrics are suitable for assessing such serial organs.

Similarly, extrapolation of the results presented here to other treatment sites should be performed with caution. By using the mean dose to quantify dosimetric accuracy, a complex three‐dimensional dose distribution has been collapsed into a single dosimetric parameter, disregarding any positional information about the dose distribution. However, this was mitigated in our study by planning all patients with the same head and neck VMAT class solution, such that the dose distributions of all 10 patients were similar. This means that the results presented here apply to the parotid and larynx when used for head and neck VMAT treatment planning. For example, although it might be expected that metrics useful for the parotid might also be useful for the rectum in prostate radiotherapy, as they are both in close proximity to a target volume and are in a region of a single steep dose gradient, further work would be required to verify this.

This study has assessed the relationship between geometric and dosimetric accuracy for several spatial metrics commonly used for assessing automatic segmentation accuracy.

Specifically, the suitability of these metrics has been assessed for the parotid and larynx in head and neck VMAT treatment planning. The results have indicated that the suitability of a spatial metric is dependent on the structure. In particular, the common volume‐based metrics, such as DSC and OI, are not related to dosimetric accuracy for the parotid, and only weakly related to dosimetric accuracy for the larynx. The surface‐based metrics were related to dosimetric accuracy and, therefore, suitable for assessing automatic segmentation accuracy for both the parotids and larynx.

## V. CONCLUSIONS

There are several spatial metrics available for assessing automatic segmentation accuracy, and there is no consensus on which metrics should be used. For treatment planning and evaluation, both geometric and dosimetric accuracy are important, as inaccurate contours can result in a suboptimal treatment plan. A suitable spatial metric should therefore be related to dosimetric accuracy. This study has measured the relationship between geometric and dosimetric accuracy for several commonly used metrics in head and neck VMAT treatment planning. We found that this relationship is structure‐dependent, and that there was no statistically significant relationship between volume‐based metrics and dosimetric accuracy for the parotids, with only a weak correlation for the larynx. The surface‐based metrics correlated with dosimetric accuracy for both structures, indicating that these metrics are more suitable measures of automatic segmentation accuracy of the parotid and larynx in the head and neck.

## COPYRIGHT

This work is licensed under a Creative Commons Attribution 4.0 International License.
